# Compound danshen dripping pills combined with trimetazidine in treating unstable angina pectoris: Protocol for a systematic review of randomized controlled trials

**DOI:** 10.1097/MD.0000000000018238

**Published:** 2019-12-10

**Authors:** Dongfeng Yao, Chuan Wang, Lihua Han, Pan Zhang, Jiping Liu, Bin Wang, Enhu Zhang

**Affiliations:** a Department of Pharmacology, College of Pharmacy, Shaanxi University of Chinese Medicine; b Key Laboratory of Pharmacodynamics and Material Basis of Chinese Medicine of Shaanxi Administration of Traditional Chinese Medicine, Xianyang, China.

**Keywords:** compound danshen dripping pills, meta-analysis, protocol, systematic review, trimetazidine

## Abstract

**Background:**

Compound danshen dripping pills (CDDP) and trimetazidine (TMZ) are commonly used in the treatment of unstable angina pectoris (UAP). Currently, the combination of CDDP and TMZ has been widely used for UAP. However, the clinical evidence CDDP combined with TMZ for treating UAP is not sufficient.

**Methods:**

We searched for randomized controlled trials testing CDDP combined with TMZ for the treatment of UAP in Chinese National Knowledge Infrastructure database, VIP database, Chinese Biological and Medicine database, Wangfang database, MEDLINE, EMBASE, Cochrane Library, and Web of Science. Extracted data are analyzed using Review Manager 5.3 software. The quality evaluation, forest plots and funnel plots will be conducted by RevMan5.3 software. Moreover, subgroup analysis and sensitivity analysis will also be completed by RevMan5.3.

**Results:**

This systematic review will provide powerful clinical evidence of combination CDDP and TMZ for treating UAP.

**Conclusions:**

This systematic review will be provided up-to-date clinical evidence to evaluate the effectiveness and safety of CDDP combined with TMZ for the treatment of parents with UAP.

**Registration Number:** PROSPERO CRD42019143100

## Abstract 2

Supplemental Digital Content is available in the text

## 1 Introduction

Coronary heart disease^[1]^ is caused by coronary artery stenosis or obstruction and can also be known as ischemic heart disease. Unstable angina pectoris (UAP) is the main manifestations of coronary heart diseases.^[2,3]^ UAP is an acute cardiac event of coronary heart disease and the intermediate clinical syndrome between chronic stable angina pectoris and acute myocardial infarction.^[1,4]^ Commonly used Western treatments for UAP include antiplatelet drugs, organic nitrates, antithrombotic drugs, and beta blockers. However, adverse reactions and drug resistance can follow such treatments.

In traditional Chinese medicine, compound danshen dripping pills (CDDPs) is a pure form of medicine which have been developed by modern high-technology means.^[5]^ The main components include *Salvia miltiorrhiza*, *Panax notoginseng*, and *Borneol*. In China, CDDP has been used for 20 years to treat CHD.^[6]^ Modern pharmacological studies have shown that CDDP has can increase coronary blood supply as well as improving microcirculation, antiplatelet aggregation and blood rheology.^[7,8]^ Trimetazidine (TMZ), a piperazine derivative, can significantly reduce myocardial oxygen consumption, optimize myocardial energy metabolism, and maintain myocardial oxygen supply balance.^[9,10]^ Myocardial energy metabolism therapy has become a new method for treating CHD, especially UAP^[11,12]^. Both CDDP and TMZ have been shown to promote myocardial metabolism and myocardial energy production. In recent years, the number of treatments developed for treating UAP has increased rapidly thanks to the integration of Chinese and Western medicine as well as randomized controlled trials (RCTs) of CDDP combined with TMZ. We therefore systematically evaluated the clinical effectiveness and safety of CDDP combined with TMZ for treating UAP.

## 2 Methods

### 2.1 Inclusion criteria

#### 2.1.1 Type of study

All of RCTs of combination of CDDP and TMZ for treating UAP should be included in this meta-analysis.

#### 2.1.2 Type of patients

According to World Health Organization^[13]^ and Chinese Cardiovascular Association,^[14]^ patients who only include UAP disease, such as other non-UAP diseases are excluded.

#### 2.1.3 Type of intervention

On the basis of taking regular western medicine, UAP patients in the experimental group take oral combination CDDP and TMZ, whereas those in the control group takes regular western medicine.

#### 2.1.4 Type of languages and regions

There are no languages or regional restrictions on the included studies. Meanwhile, we will search studies until May 2019.

#### 2.1.5 Type of outcomes

The clinical effectiveness and electrocardiogram improvement are the primary outcome measurements. Among these primary outcomes, the clinical effectiveness is defined as >50% reduction in frequency of angina attacks and weekly frequency of angina attacks reduction. Additional outcomes include major adverse cardiovascular events, QT interval dispersion, corrected QT interval dispersion, ST-segment depression, stroke volume, ejection fraction, and total ischemia burden. Finally, we will also assess the incidence of adverse events.

### 2.2 Search strategy

#### 2.2.1 Study search

Two researchers will independently search for RCTs which evaluate CDDP and TMZ for treating UAP in the China National Knowledge Infrastructure Database, Wanfang Database, Chinese Scientific Journals Database, Chinese Biomedical Literature Database, Medline, Cochrane Library, Web of Science, and other databases and from inception until May 2019.

#### 2.2.2 Search for the specific strategies

These main keywords in database are “compound Danshen dripping pills” AND “unstable angina pectoris” OR “coronary heart disease” OR “angina pectoris” [Title/Abstract], “trimetazidine” AND “unstable angina pectoris” OR “coronary heart disease” OR “angina pectoris” [Title/Abstract], “compound Danshen dripping pills” AND “trimetazidine” AND “unstable angina pectoris” OR “coronary heart disease” OR “angina pectoris” [Title/Abstract]. The search strategy of PubMed will be shown in supplemental digital Appendix A.

#### 2.2.3 Searching for other resources

In addition, we will retrieve the conference reports and contact experts not included in the above those databases and connect the authors to obtain important information.

### 2.3 Data extraction and analysis

#### 2.3.1 Selection of studies

**Figure 1:**
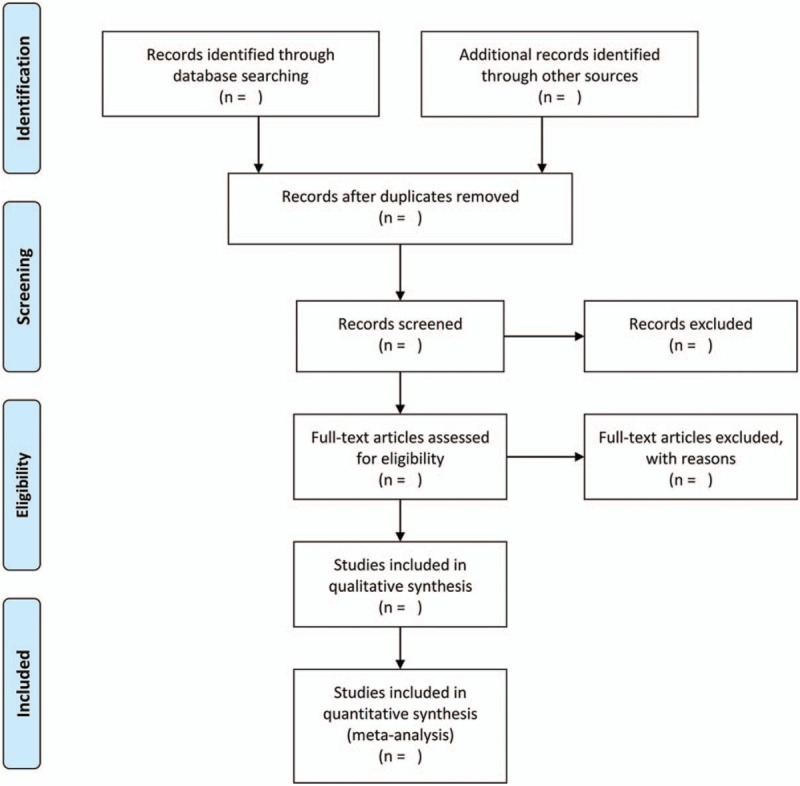
The selection of studies in flow diagram.

Two researchers search databases independently, and then included studies are downloaded into Endnote software (version X8, Thomson Reuters, Inc., New York, NY) to next selection. Through Endnote software, exclude duplicate studies. Full-text studies will be performed while the title/abstract thought to be thematic. Finally, we should exclude some studies for reasons, such as animal work, review, inclusion criteria, and so on. Details of selection of articles are shown in PRISMA flowchart (Fig. 1).

#### 2.3.2 Data extraction

Through a series of selection, the data of included RCTs are extracted. Two researchers collected data including number of patients, patients’ age, course of treatment, stage, interventions details, outcomes, and adverse events. When there will be differences, consensus is reached through discussion. If necessary, the third researcher will be invited to participate in the discussion. If the data are missing or not clear, we will contact the author of this article to explain.

#### 2.3.3 Heterogeneity analysis

The odds ratio will be results of dichotomous variables, with 95% confidence intervals (CIs 95%). And continuous variables will be measured by mean difference, with 95% CIs. The Q statistic and *I*^2^ are used to analyze heterogeneity among the studies. If *P* > .1 and *I*^2^ ≤ 50%, the fixed effect model is used for meta-analysis; if *P* ≤ .1 and *I*^2^ > 50%, the random effect model is used for meta-analysis. A *P* value of <0.05 for differences between groups is considered statistically significant.

### 2.4 Risk of bias

The quality assessment is conducted independently by 2 researchers and the final results are determined through discussion in the event of any disagreement. Selected literature can be divided into 7 considerations to evaluate the risk of bias, following the recommendations of the Cochrane Handbook: random sequence generation method, allocation concealment, blinding of participants and personnel, blinding of outcome assessment, incomplete outcome data, selective reporting, and other offset sources. Each consideration is divided into 3 levels: “low risk,” “high risk,” and “unclear.”

### 2.5 Publication bias

When more than 10 studies are included this index, we should make a funnel plot to analyze whether it is symmetric. Meanwhile, here are some other methods such as Begg rank correlation test and Egger linear regression test to evaluate publication bias.

### 2.6 Subgroup analysis

Subgroup analysis will be used to detect the source of heterogeneity. We will conduct subgroup analysis mainly from the drug dose of intervention, treatment cycle, age stage difference, and quality evaluation results of included RCTs.

### 2.7 Sensitivity analysis

Sensitivity analysis is an important method to evaluate the robustness and reliability of meta-analysis. For the sensitivity analysis, we will gradually screen out the included studies one by one, so as to find out the studies that have a serious impact on the results of meta-analysis.

## 3 Discussion

UAP, as an important global health problem, has seriously affected the quality of life. The search for a treatment with UAP is imminent.^[15]^ As a new medical treatment, complementary therapy has a certain effect on the treatment of coronary artery disease.^[16]^ This protocol will show up-to-date complementary therapy of combination of CDDP and TMZ for the treatment of UAP, and through this therapy to evaluate clinical efficacy and safety for UAP treatment. Meanwhile, this meta-analysis will provide evidence-based medicine for UAP treatment. Nevertheless, the safety of CDDP combined with TMZ for UAP will be evaluated, so we should discreetly treat the results of systematic evaluation.

## Author contributions

**Formal analysis:** Dongfeng Yao, Pan Zhang, Lihua Han.

**Methodology:** Dongfeng Yao, Jiping Liu, Pan Zhang.

**Conceptualization:** Chuan Wang, Enhu Zhang.

**Data curation:** Dongfeng Yao, Chuan Wang, Jiping Liu.

**Formal analysis:** Dongfeng Yao, Lihua Han.

**Funding acquisition:** Chuan Wang.

**Methodology:** Dongfeng Yao, Pan Zhang.

**Software:** Dongfeng Yao.

**Supervision:** Chuan Wang, Bin Wang.

**Writing – original draft:** Dongfeng Yao.

**Writing – review and editing:** Dongfeng Yao, Chuan Wang.

Chuan Wang orcid: 0000-0002-8016-0113.

## Supplementary Material

Supplemental Digital Content

## References

[1] Nwafor IA Eze JC Surgical management of CHD in the adult population: the role of humanitarian cardiac surgery mission in Nigeria. Cardiol Young 2019;29:11–5. 30033908 10.1017/S1047951118000793

[2] Song H Wang P Liu J Panax notoginseng preparations for unstable angina pectoris: a systematic review and meta-analysis. Phytother Res 2017;31:1162–72. 28634988 10.1002/ptr.5848

[3] Gao Z Wei B Qian C Puerarin injection for treatment of unstable angina pectoris: a meta-analysis and systematic review. Int J Clin Exp Med 2015;8:14577–94. 26628941 PMC4658830

[4] Zhang D Wu J Liu S Salvianolate injection in the treatment of unstable angina pectoris: a systematic review and meta-analysis. Medicine (Baltimore) 2016;95:e5692. 28002341 10.1097/MD.0000000000005692 PMC5181825

[5] Lv C Liu C Liu J The effect of compound danshen dripping pills on the dose and concentration of warfarin in patients with various genetic polymorphisms. Clin Ther 2019;41:1097–109. 31053296 10.1016/j.clinthera.2019.04.006

[6] Writing Group of Recommendations of Expert Panel from Chinese Geriatrics Society on the Clinical Use of Compound Danshen Dripping Pills. Recommendations on the clinical use of compound danshen dripping pills. Chin Med J (Engl) 2017;130:972–8. 28397728 10.4103/0366-6999.204106 PMC5407045

[7] Zhou W Song XG Chen C Study on action mechanism and material base of compound Danshen dripping pills in treatment of carotid atherosclerosis based on techniques of gene expression profile and molecular fingerprint [in Chinese]. Zhongguo Zhong Yao Za Zhi 2015;40:3308–13. 26790312

[8] Zhou W Yuan WF Chen C Study on material base and action mechanism of compound danshen dripping pills for treatment of atherosclerosis based on modularity analysis. J Ethnopharmacol 2016;193:36–44. 27396350 10.1016/j.jep.2016.07.014

[9] Su Q Li L Zhao J Effects of trimetazidine on periprocedural microRNA-21 expression by CD4+ T lymphocytes in patients with unstable angina pectoris. Oncotarget 2017;8:104992–9. 29285227 10.18632/oncotarget.20975 PMC5739614

[10] Qiu ZX Ma HJ Wang DF Observation on effect of compound danshen droplet-pill combined with trimetazidine in treating senile unstable angina pectoris [in Chinese]. Zhongguo Zhong Xi Yi Jie He Za Zhi 2005;25:787–9. 16248238

[11] Katano Y Takeda K Otorii T Effects of dilazep on cardiac function, coronary circulation and myocardial energy metabolism [in Japanese]. Nihon Yakurigaku Zasshi 1974;70:305–14. 4858949

[12] Wang S Ye L Wang L Protective mechanism of shenmai on myocardial ischemia-reperfusion through the energy metabolism pathway. Am J Transl Res 2019;11:4046–62. 31396317 PMC6684917

[13] Nomenclature, criteria for diagnosis of ischemic heart, disease. Report of the Joint International Society and Federation of Cardiology/World Health Organization task force on standardization of clinical nomenclature. Circulation 1979;59:607–9. 761341 10.1161/01.cir.59.3.607

[14] Chinese Society of Cardiology, Editoral Committee on Chinese Journal of Cardiology. Recommendations for the diagnosis and treatment of unstable angina pectoris. Chin J Cardiol 2000;8:409–12.

[15] Kusumoto FM Schoenfeld MH Barrett C 2018 ACC/AHA/HRS guideline on the evaluation and management of patients with bradycardia and cardiac conduction delay: a report of the American College of Cardiology/American Heart Association Task Force on Clinical Practice Guidelines and the Heart Rhythm Society. Heart Rhythm 2019;16:e128–226. 30412778 10.1016/j.hrthm.2018.10.037

[16] Iqbal J Widmer R Gersh BJ State of the art: optimal medical therapy—competing with or complementary to revascularisation in patients with coronary artery disease? EuroIntervention 2017;13:751–9. 28844035 10.4244/EIJ-D-17-00463

