# Supplementary material for: Compound danshen dripping pills combined with trimetazidine in treating unstable angina pectoris: Protocol for a systematic review of randomized controlled trials
Source: Medicine (Baltimore). 2019 Dec 10;98(49):e18238. doi: 10.1097/MD.0000000000018238 (PMC6919410; doi:10.1097/MD.0000000000018238)
Supplement: Supplemental Digital Content [file medi-98-e18238-s001.docx]

**Supplementary Material**

**Search strategy used in PubMed database**

#1 unstable angina OR Anginas, Unstable OR Angina Pectoris, Unstable OR Angina Pectori, Unstable OR Unstable Angina Pectori OR Unstable Angina Pectoris.

#2 compound Danshen dripping pills OR CDDP OR CP OR fufang danshen pills OR fufang danshen diwan OR danshen diwan OR danshen pills.

#3 Trimetazidine OR TMZ OR C_14_H_22_N_2_O_3_ (Molecular structure) OR 1-(2,3,4-trimethoxybenzyl) piperazine OR Trimetazidine hydrochloride tablets OR Trimetazidine tablets OR wanshuangli OR wanshuangli tablets.

#4 Randomized controlled trial OR clinical study OR Clin-ical Trial OR Controlled study OR Controlled Trial OR Random*Control* study OR random* Control* Trial

#1 AND #2 AND #3 AND #4
